# Proteomic analysis of transcutaneous auricular vagus nerve stimulation induced cognitive restoration in a mouse model of vascular cognitive impairment

**DOI:** 10.1186/s13041-026-01339-6

**Published:** 2026-07-25

**Authors:** Seunghwan Choi, Jae Hyun Park, Chan Su Kim, Sun Kwang Kim

**Affiliations:** 1 Department of Physiology, College of Korean Medicine, Kyung Hee University, Seoul, 02447 Korea; 2 Department of East-West Medicine, Graduate School, Kyung Hee University, Seoul, 02447 Korea

**Keywords:** Transcutaneous auricular vagus nerve stimulation, Vascular cognitive impairment, Proteomic analysis, TrkB

## Abstract

Vascular cognitive impairment (VCI) is a major cause of dementia with limited treatment options. Transcutaneous auricular vagus nerve stimulation (taVNS) is a noninvasive neuromodulation approach that has shown cognitive benefits, but its molecular basis remains unclear. Here, we employed a mouse model of transient bilateral common carotid artery occlusion (tBCCAO) to identify molecular candidates underlying the cognitive restorative effects of taVNS. tBCCAO impaired cognitive function as revealed by behavioral assessments, whereas taVNS restored behavioral performance. Hippocampal proteomic analysis identified 58 proteins altered in tBCCAO compared with sham, and 16 proteins significantly modulated by taVNS compared with tBCCAO. Among these, a core set of taVNS-responsive proteins exhibited a normalization-like pattern, suggesting that taVNS shifted ischemia-associated proteomic alterations toward a near-normal state rather than simply amplifying or suppressing the overall response. These findings suggest that taVNS may improve VCI by restoring hippocampal protein dysregulation following transient cerebral hypoperfusion and highlight molecular targets as candidate mechanisms for further investigation.

## Main text

Vascular cognitive impairment (VCI) refers to a multifactorial spectrum of cognitive deficits resulting from cerebrovascular disease or vascular factors [1]. VCI is the second most prevalent type of dementia following Alzheimer’s disease, accounting for 15%–30% of all dementia cases [2]. Although cognitive enhancers such as donepezil, rivastigmine, and memantine are approved, their benefits are modest and often require high doses [3]. Considering the side effects associated with high-dose usage and the limited efficacy, there is a pressing need for novel therapeutic strategies for VCI that do not rely solely on pharmacological approaches.

Transcutaneous auricular vagus nerve stimulation (taVNS) is a noninvasive neuromodulation technique that stimulates the sensory branch of the vagus nerve in the ear, thereby activating the brain via the nucleus of the solitary tract [4]. taVNS has been reported to induce significant recovery of cognitive function in both preclinical animal models of VCI and clinical trials involving patients with mild cognitive impairment [5, 6]. Given that taVNS exerts multimodal effects across the brain, identifying a single molecular mediator of cognitive restoration remains elusive. In this study, therefore, we employed a proteomic approach to identify candidate molecular targets associated with taVNS.

**Fig. 1:**
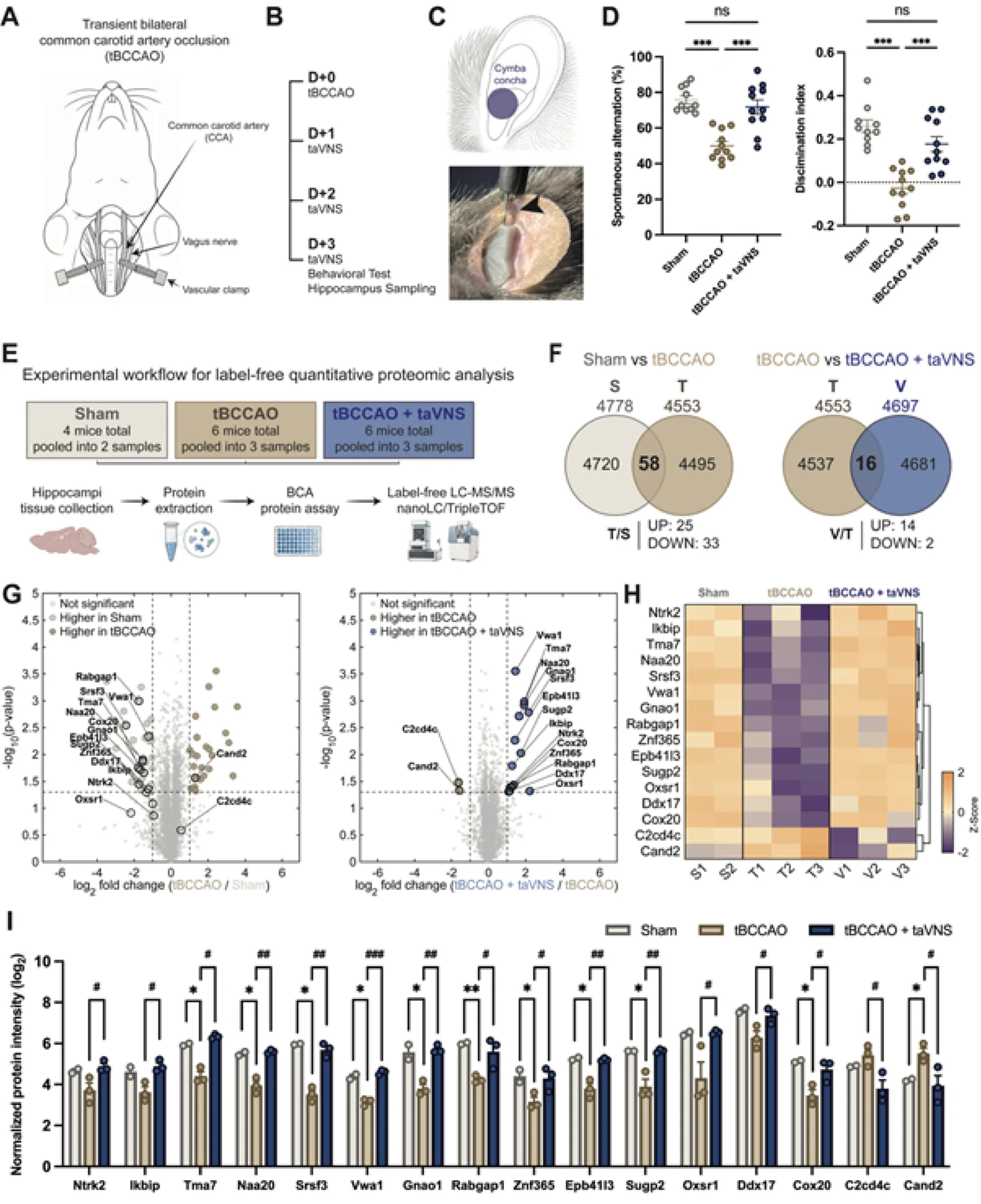
taVNS restores tBCCAO-induced cognitive impairment and proteomic analysis identifies hippocampal proteomic targets underlying recovery. **A** Schematic illustration of tBCCAO. **B** Experimental timeline. tBCCAO was performed on day 0, followed by taVNS treatment for three consecutive days. Behavioral testing and hippocampal tissue sampling were performed on day 3 in separate cohorts subjected to the same tBCCAO and taVNS protocol. **C** Representative images showing target area of cymba concha and taVNS electrode placement. Blue circle marks cymba concha. Arrowhead indicates the stimulation electrode connected to the custom-made stimulus apparatus. **D** Behavioral assessment for cognitive function using the Y-maze and novel object recognition test. Statistical analysis was performed using one-way ANOVA followed by Tukey’s multiple comparisons test. ***p < 0.001; ns, not significant. Data are represented as mean ± SEM. **E** Experimental workflow for label-free quantitative proteomic analysis. Hippocampal tissues from sham, tBCCAO, and tBCCAO + taVNS mice were pooled in each group, followed by protein extraction, BCA assay, and LC–MS/MS analysis. **F** Venn diagrams showing quantified proteins and DEPs in the sham vs tBCCAO comparison (T/S) and the tBCCAO vs tBCCAO + taVNS comparison (V/T). DEPs were defined by p ≤ 0.05 and fold change ≥ 2 or ≤ 0.5. **G** Volcano plots showing hippocampal proteomic changes in the tBCCAO/sham (left) and tBCCAO + taVNS/tBCCAO comparisons (right). Selected V/T significant proteins are labeled with bold circles **H** Heatmap visualization of the 16 V/T significant DEPs using z-scored normalized log₂ protein intensities across individual samples. Hierarchical clustering was performed on protein rows, and z-scores were capped at ± 2 for visualization. **I** Quantitative comparison of normalized log₂ protein intensities for the selected V/T significant DEPs. Statistical analysis was performed using Student's t-test. **p < 0.01; *p < 0.05 (sham vs tBCCAO); ###*p* < 0.001; ##*p* < 0.01; #*p* < 0.05 (tBCCAO vs tBCCAO + taVNS). Data are represented as mean ± SEM.

To this end, VCI was induced in 8-week-old male C57BL/6 mice (Koatech, Pyeongtaek, Korea) by transient bilateral common carotid artery occlusion (tBCCAO). For this, both common carotid arteries were exposed with the vagus nerve preserved and clipped for 25 min before reperfusion under isoflurane anesthesia (Fig. 1A). Beginning the day after surgery, taVNS was delivered under isoflurane anesthesia to the left cymba concha and dorsal auricle for three consecutive days via a custom-made electrodes connected to a Digistim2 generator (ALA Scientific Instruments, Farmingdale, NY, USA), applying rectangular bipolar pulses at 2 Hz and 5 V for 20 min per session (Fig. 1B, C). 2 Hz was selected based on our previous finding that the low frequency of 2 Hz taVNS promotes cerebral vasomotion and cerebrospinal fluid influx associated with brain clearance effects [7]. On day 3, following the final taVNS session, cognitive function was assessed using the Y-maze test and novel object recognition test.

In the Y-maze test, the tBCCAO group showed a significant decrease in spontaneous alternation behavior compared with the sham group (Fig. 1D, left; *p* < 0.001), whereas taVNS restored behavioral performance (*p* < 0.001). Similarly, in the novel object recognition test, the discrimination index was markedly reduced in the tBCCAO group but recovered in tBCCAO + taVNS (Fig. 1D, right; *p* < 0.001). Consistent with previous studies, these results demonstrate that taVNS restored impaired cognitive function [5, 8].

To identify the underlying molecular changes, a separate cohort of mice underwent the same tBCCAO and taVNS protocol, and the hippocampus was harvested immediately after the final taVNS session. Hippocampal tissue was then pooled within each group (sham, n = 2 pools from 4 mice; tBCCAO, n = 3 pools from 6 mice; tBCCAO + taVNS, n = 3 pools from 6 mice), and proteins were extracted and quantified by BCA assay prior to label-free quantitative LC–MS/MS on a nanoLC/TripleTOF platform (E-biogen, Korea) (Fig. 1E). Proteins were identified and quantified with DIA-NN (v1.8) against the SwissProt Mouse 2022.01 database (precursor and protein-group q-value ≤ 0.01), and differentially expressed proteins (DEPs) were defined in Perseus (v1.6.15.0) as *p* ≤ 0.05 with a fold change ≥|2|.

Approximately 4,500–4,800 proteins were consistently quantified following preprocessing and QC filtering from each sample. LC–MS/MS-based comparative analysis was performed between tBCCAO and sham (T/S) and between taVNS and tBCCAO (V/T) to characterize transient hypoperfusion-induced proteomic changes and taVNS-induced modulation. In the T/S comparison, 58 DEPs were identified, with 25 upregulated and 33 downregulated. Similarly, in the V/T comparison, 16 DEPs were identified, with 14 upregulated and 2 downregulated (Fig. 1F). Together, these findings indicate significant and distinct proteomic changes following tBCCAO and taVNS.

Interestingly, the analysis pinpointed a distinct subset of proteins whose pathological dysregulation following ischemic insult was shifted toward normalization by taVNS. Volcano plots revealed that a panel of proteins upregulated by taVNS, including Ntrk2, Ikbip, Tma7, Naa20, Srsf3, Vwa1, Gnao1, Rabgap1, Znf365, Epb41l3, Sugp2, Oxsr1, Ddx17, and Cox20, were reciprocally downregulated following tBCCAO (Fig. 1G, left), whereas C2cd4c and Cand2, which were downregulated by taVNS, were conversely upregulated by tBCCAO (Fig. 1G, right). This regulation pattern suggests that taVNS induced a normalization‑like shift, rather than merely dampening the overall response. We next examined the core 16 DEPs significantly altered in the V/T comparison. Heatmap analysis showed coordinated protein modules with a normalization-like pattern (Fig. 1H). Quantitative examination at the individual protein level further supported this restoration. 14 proteins significantly decreased by tBCCAO were restored toward sham levels following taVNS treatment, while 2 proteins displayed the opposite regulatory pattern (Fig. 1I).

Our results demonstrate that taVNS attenuates hippocampal protein dysregulation induced by transient cerebral ischemia, together with restoration of cognitive performance. Among the taVNS-responsive proteins, we highlight TrkB (Tropomyosin receptor kinase B) encoded by *Ntrk2* (Neurotrophic receptor tyrosine kinase 2) as a particularly noteworthy molecule. TrkB is the primary receptor for BDNF (Brain-derived neurotrophic factor) and is closely linked to neurotrophic signaling, synaptic plasticity, and neuronal survival [9]. This is consistent with previous reports that VNS engages BDNF–TrkB signaling and that its therapeutic effects may be mediated through TrkB-dependent mechanisms [10–12]. Taken together, our findings provide molecular candidates linking taVNS to cognitive restoration in VCI and offer a basis for further mechanistic investigation.

## Data Availability

All data supporting the findings of this study are available from the corresponding author upon reasonable request.

## Abbreviations

VCI: Vascular cognitive impairment
taVNS: Transcutaneous auricular vagus nerve stimulation
tBCCAO: Transient bilateral common carotid artery occlusion
CCA: Common carotid artery
DEP: Differentially expressed protein
TrkB: Tropomyosin receptor kinase B
Ntrk2: Neurotrophic receptor tyrosine kinase 2
BDNF: Brain-derived neurotrophic factor
